# Comparative genomics analysis of *Streptococcus iniae* isolated from *Trachinotus ovatus*: novel insight into antimicrobial resistance and virulence differentiation

**DOI:** 10.1186/s12864-023-09882-5

**Published:** 2023-12-14

**Authors:** Xiangying Xiong, Ruifang Chen, Junxiang Lai

**Affiliations:** 1 Guangxi Key Laboratory of Marine Environmental Science, Guangxi Academy of Marine Sciences, Guangxi Academy of Sciences, 98 Daling Road, Nanning, 530007 Guangxi China; 2 Guangxi Institute of Oceanology Limited Liability Company, Beihai, 536000 Guangxi China; 3 Beibu Gulf Marine Industry Research Institute, Fangchenggang, 538000 Guangxi China

**Keywords:** *Streptococcus iniae*, Comparative genomics, Antibiotic resistance, Virulence

## Abstract

**Background:**

*Streptococcus iniae* is an important fish pathogen that cause significant economic losses to the global aquaculture industry every year. Although there have some reports on the genotype of *S.iniae* and its relationship with virulence, no genome-scale comparative analysis has been performed so far. In our previous work, we characterized 17 isolates of *S.iniae* from *Trachinotus ovatus* and divided them into two genotypes using RAPD and rep-PCR methods. Among them, BH15-2 was classified as designated genotype A (in RAPD) and genotype 1 (in rep-PCR), while BH16-24 was classified as genotype B and genotype 2. Herein, we compared the differences in growth, drug resistance, virulence, and genome between BH15-2 and BH16-24.

**Results:**

The results showed that the growth ability of BH16-24 was significantly faster than that of BH15-2 at the exponential stage. Antimicrobial tests revealed that BH15-2 was susceptible to most of the tested antibiotics except neomycin and gentamycin. In contrast, BH16-24 was resistant to 7 antibiotics including penicillin, sulfasomizole, compound sulfamethoxazole tablets, polymyxin B, spectinomycin, rifampin and ceftazidime. Intraperitoneal challenge of *T.ovatus*, showed that the LD_50_ value of BH15-2 was 4.0 × 10^2^ CFU/g, while that of BH16-24 was 1.2 × 10^5^ CFU/g. The genome of *S.iniae* BH15-2 was 2,175,659 bp with a GC content of 36.80%. Meanwhile, the genome of BH16-24 was 2,153,918 bp with a GC content of 36.83%. Comparative genome analysis indicated that compared with BH15-2, BH16-24 genome had a large-scale genomic inversion fragment, at the location from 502,513 bp to 1,788,813 bp, resulting in many of virulence and resistance genes differentially expression. In addition, there was a 46 kb length, intact phage sequence in BH15-2 genome, which was absent in BH16-24.

**Conclusion:**

Comparative genomic studies of BH15-2 and BH16-24 showed that the main difference is a 1.28 Mbp inversion fragment. The inversion fragment may lead to abnormal expression of drug resistant and virulence genes, which is believed to be the main reason for the multiple resistance and weakened virulence of BH16-24. Our study revealed the potential mechanisms in underlying the differences of multidrug resistance and virulence among different genotypes of *S.iniae*.

**Supplementary Information:**

The online version contains supplementary material available at 10.1186/s12864-023-09882-5.

## Background

*Streptococcus iniae* is a prominent warm-water pathogen and is known to infect a wide range of fish species. The infected fish exhibit varied clinical symptoms of erratic swimming, lethargy, meningoencephalitis, exophthalmia, enteritis and septicaemia [1, 2]. According to the previous reports, *S.iniae* can cause severe diseases in *Trachinotus ovatus* and result significant economic losses [3, 4]. Meanwhile, *S.iniae* is a zoonotic bacteria that poses a threat to public health [5].

Different from other streptococci of the same genus, the serotype of *S.iniae* cannot be classified by the traditional method of Lancefield [6]. Random amplified polymorphic DNA (RAPD) or repetitive primer polymerase chain reaction (rep-PCR) method can be used to distinguish the serotypes of *S.iniae* [7, 8]. Through analysis of rep-PCR and RAPD, 29 isolates of *S.iniae* were divided into two genotypes and a correlation between the genotype and the virulence had been identified [9]. Fuller et al. reported that *S.iniae* virulence is associated with distinct genetic profile and demonstrated differences between pathogenic and nonpathogenic isolates [10]. Rep-PCR analysis of 14 *S.iniae* strains from diseased *O.niloticus* presented genetic heterogeneity and were divided into six genotypes banding patterns [11]. The routine methods for preventing bacterial infections are the use of antibiotics and chemotherapeutants, but antibiotic usage in the aquaculture industry is largely uncontrolled. Abuse and/or excessive use of antibiotics can lead to the emergence of antimicrobial-resistant bacteria [12]. However, there is little information about the mechanisms behind antibiotic resistance and virulence differentiation among diverse *S.iniae* strains, with the lack of information hampering the effective treatment of the disease.

Comparative genomics analysis is a helpful way to identify genome-wide genetic variants of bacteria that may be associated with host and geographic origin but also to better understand their potential pathogenicity and antibiotic resistance. Comparative genomic analysis of *S. agalactiae* isolates with distinct clinical origins or host associations has provided insight into potential mechanisms of evolution, virulence, and host adaptation [13]. Fanelli et al. identified a large number of antibiotic, heavy metals and virulence determinants by reporting the whole genome sequencing and genomic characterization of two *Arcobacter butzleri* strains isolated from shellfish [14]. Similarly, the virulence and antimicrobial resistance-associated genomic determinants of two *Salmonella* Typhimurium strains were reported through comparative genomics approaches [15]. Although some complete genome sequences of *S.iniae* isolated have been sequenced, to our knowledge, there have been no reports on the comparative genomics analysis for understanding the genetic basis of pathogenicity and multidrug resistance. In our previous work, we isolated 17 strains of *S.iniae* from *T.ovatus* and differentiated them into two genotypes using RAPD and rep-PCR methods [16]. Among them, BH15-2 was divided as designated genotype A in RAPD analysis and genotype 1 in rep-PCR analysis, while BH16-24 was classified as genotype B and genotype 2. Here, we compared the phenotypes (growth, drug-resistance, and virulence variation) and genome of BH15-2 and BH16-24, which may explain the mechanism of their differences in biological properties.

## Results

### Clinical symptoms of infected fish and biochemical characterization of the strain

**Fig. 1:**
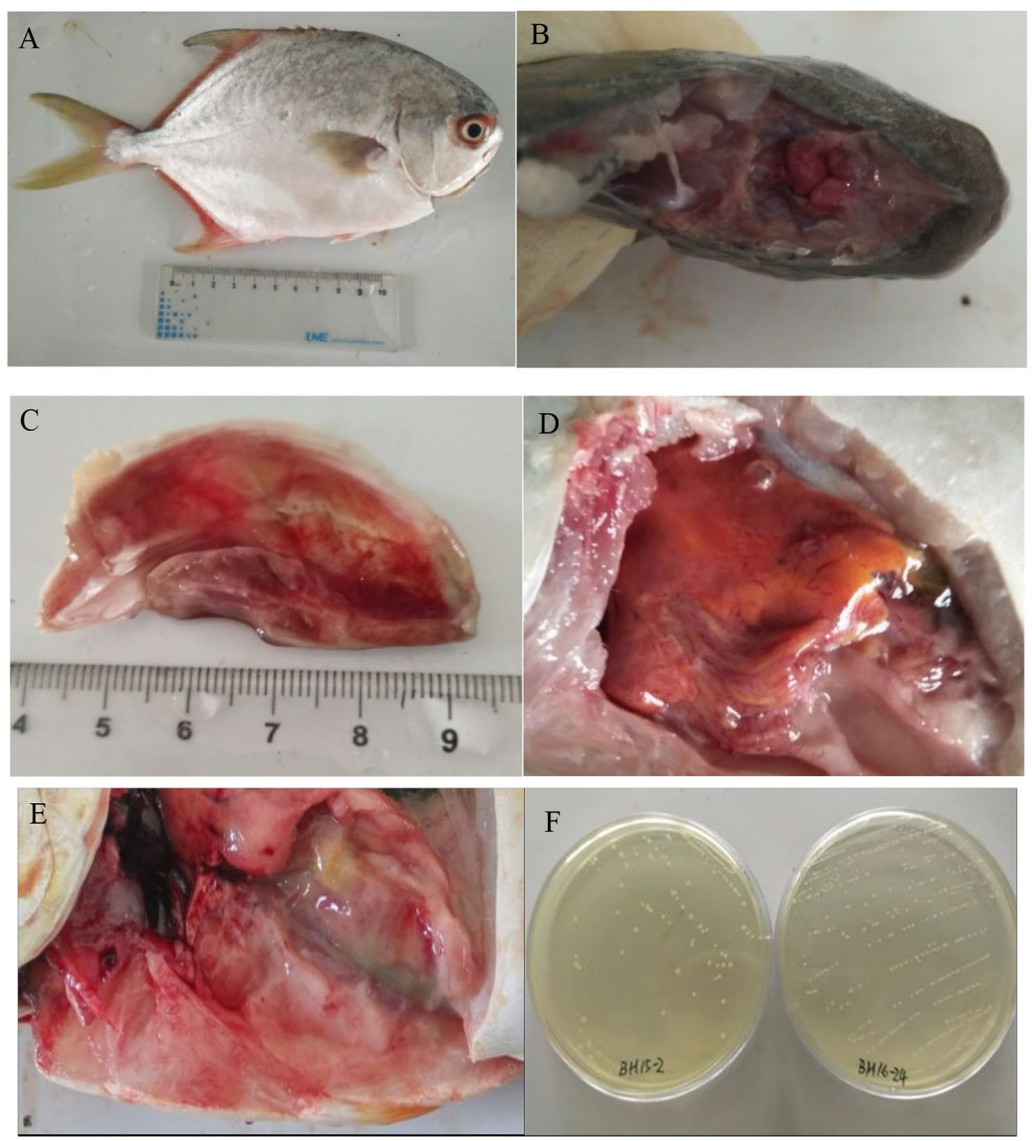
Clinical signs of diseased *T.ovatus*. (**A**) pterygiophore hemorrhage, (**B**) meningoencephalitis, (**C**) operculum hemorrhage (**D**) liver hemorrhage (**E**) enteritis (**F**) Bacterial colony on BHI agar

**Fig. 2:**
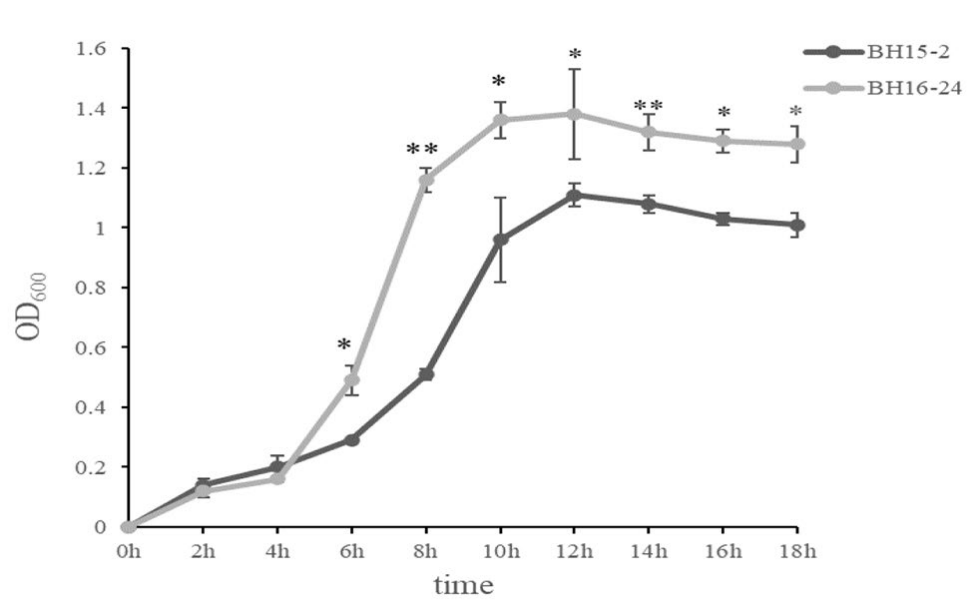
Growth curve of BH15-2 and BH16-24 strains cultured in BHI broth Aliquots of cell culture were taken at various time points and measured for cell density at OD_600,_ *indicates *P* < 0.05,**indicates *P* < 0.01

In this study, the natural infected fish were observed with symptoms of meningoencephalitis, enteritis, and hemorrhage of pterygiophore, operculum and liver (Fig. 1A-E). After 24 h incubation at 28 ℃ on BHI agar plates, the pathogenic bacteria formed circular, buff, smooth surface, intact edge, convex colonies with a diameter of approximately 0.5-1.0 mm (Fig. 1F). Gram staining showed that the strains are Gram-positive coccus appearing in short chains under an optical microscope. Biochemical analyses revealed complete homogeneity in BH15-2 and BH16-24. According to the growth curves, the growth rate of BH16-24 was significantly faster than that of BH15-2 after 6 h of culture (*p* < 0.05) (Fig. 2).

### The results of virulence test

The infection results from BH15-2 and BH16-24 were shown in Table 1. Cumulative mortality rates of BH15-2 and BH16-24 after post infection were illustrated in Table 2. At 28 days post-infection (dpi), BH15-2 induced higher levels of mortality and revealed a greater virulence than BH16-24. Concretely, the LD_50_ value of BH15-2 was 4.0 × 10^2^ CFU/g, while that of BH16-24 was 1.2 × 10^5^ CFU/g (body weight). The virulence of BH15-2 was 1000 times than BH16-24. BH15-2 caused 75%, 75%, 87.5% and 100% cumulative mortality, while BH16-24 caused 6.25%, 6.25%, 12.5% and 62.5% at 1.5 × 10^5^, 1.5 × 10^6^, 1.5 × 10^7^, 1.5 × 10^8^ cfu/ml concentrations in *T. ovatus* respectively. In the control groups, there was no mortality during the observation period. Most deaths occurred within 48 h p.i. and did not show any clinical signs of disease prior to death. But bacterial isolated were retrieved from the brain and liver of the challenged fish and found to be the same species.

**Table 1:** Total mortality of *T. ovatus* challenged by intraperitoneal injection with 100 µl of *S.iniae* strains BH15-2 and BH16-24

Group	Bacterial concentration cfu/ml	No. of fish per group	No. of mortalities	Total mortality %
BH15-2	1.5 × 10^8^	16	16	100
	1.5 × 10^7^	16	14	87.5
	1.5 × 10^6^	16	12	75
	1.5 × 10^5^	16	12	75
BH16-24	1.5 × 10^8^	16	10	62.5
	1.5 × 10^7^	16	2	12.5
	1.5 × 10^6^	16	1	6.25
	1.5 × 10^5^	16	1	6.25
Control	Physiological saline	16	0	0

**Table 2:** Results of antimicrobial susceptibility of BH15-2 and BH16-24.

Drug	Sensitivity criteria of R, I, S			Inhibition zone (mm)		Drug	Sensitivity criteria of R, I, S			Inhibition zone (mm)	
	R	I	S	BH15-2	BH16-24		R	I	S	BH15-2	BH16-24
β-lactams						Aminoglycosides					
Penicillin(10U)	≤ 19	20–27	≥ 28	44 (S)	10 (R)	Neomycin(30)	≤ 16	17–23	≥ 24	22 (I)	19 (I)
Ampicilin(10)	≤ 13	14–16	≥ 17	28 (S)	14 (I)	Gentamycin(10)	≤ 15	16–23	≥ 24	17 (I)	18 (I)
Ceftazidime(30)	≤ 17	18–20	≥ 21	30 (S)	8 (R)	Spectinomycin(100)	≤ 14	15–18	≥ 19	19 (S)	11 (R)
Cefazolin(30)	≤ 10	10–20	≥ 20	46 (S)	14 (I)	Macrolides					
Ceftriaxone Sodium(30)	≤ 13	14–22	≥ 23	42 (S)	15 (I)	Erythromycin(15)	≤ 13	14–22	≥ 23	32 (S)	27 (S)
Sulfonamides						Azithromycin(15)	≤ 13	14–17	≥ 18	20 (S)	19 (S)
Sulfasomizole(300)	≤ 12	13–16	≥ 17	34 (S)	0 (R)	Quinolones					
Compound Sulfamethoxazole(1.25/23.75)	≤ 10	11–15	≥ 16	24 (S)	0 (R)	Ciprofloxacin(5)	≤ 15	16–20	≥ 21	38 (S)	25 (S)
Tetracyclines						Norfloxacin(10)	≤ 12	13–16	≥ 17	21 (S)	22 (S)
Tetracycline(30)	≤ 14	15–18	≥ 19	32 (S)	22 (S)	Chloramphenicol					
Deoxytetracycline(30)	≤ 17	18–22	≥ 23	36 (S)	24 (S)	Chloramphenicol(30)	≤ 12	13–17	≥ 18	34 (S)	24 (S)
Rifamycin						Polypeptide					
Rifampin(5)	≤ 16	17–19	≥ 20	35 (S)	14 (R)	Polymyxin B(300U)	≤ 8	9–11	≥ 12	13 (S)	0 (R)

Note: S: susceptible; I: intermediately susceptible; R: resistant

### Drug resistance

The antibiogram study results are shown in Table 2. Both strains were sensitive to tetracycline, erythromycin, norfloxacin, azithromycin, ciprofloxacin, chloramphenicol, deoxytetracycline, and intermediate resistant toward to neomycin, gentamycin. BH16-24 was resistant or intermediate resistant toward all the β-lactam antibiotics tested in this study, i.e., penicillin, ampicilin, cefazolin, ceftriaxone Sodium, and ceftazidime, while BH15-2 was sensitive to these antibiotics. In addition, BH16-24 was resistant to sulfonamide antibiotics (sulfasomizole, compound sulfamethoxazole), polymyxin B, aminoglycoside antibiotic (spectinomycin), rifampin, whereas BH15-2 was susceptibility to these antibiotics. Therefore, BH16-24 is a multidrug-resistant (MDR) bacterium. The calculated MAR index of BH15-2 was 0, while BH16-24 was 0.37.

### Comparative Genomics

#### General features of whole genome sequencing results

**Fig. 3:**
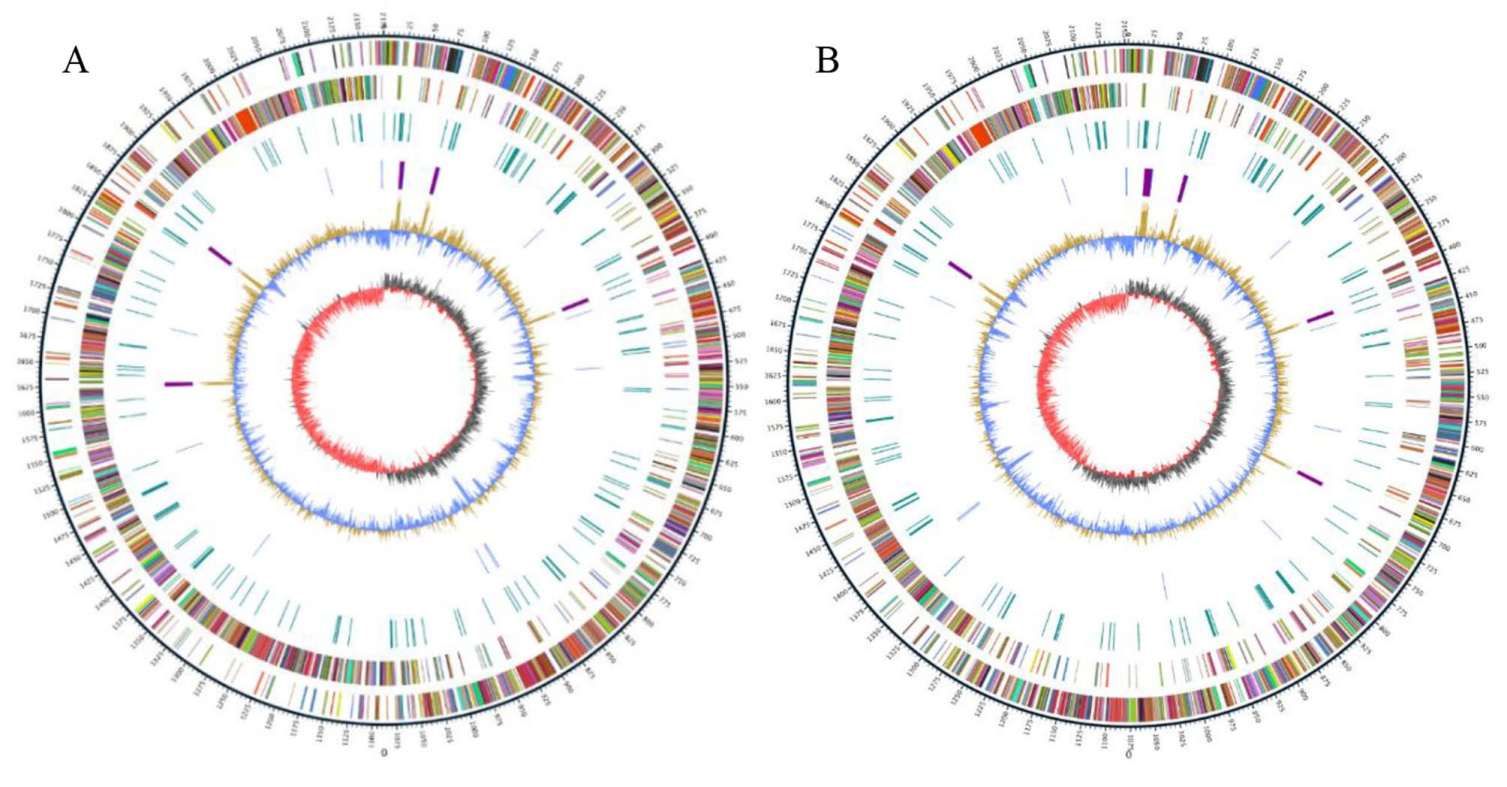
Schematic circular diagrams of the *S.iniae* BH15-2 (**A**) and BH16-24 (**B**) genomes Circle 1 (from outside to inside): scale (in kb); circles 2 and 3: genes in positive strand and negative strand; circle 4: repetitive sequences; circle 5: tRNA (in blue) and rRNA (in purple); circles 6: GC content; circles 7: GC skew

The general features of BH15-2 and BH16-24 genomes are summarized in Table 3. The genome size of BH15-2 was 2,175,659 bp with a GC content of 36.80%, while BH16-24 is 2,153,918 bp long with a GC content of 36.83%. Both genomes were optimized to assemble a circular genome with 0 gap. The genome of BH15-2 contained 2090 genes, 60 tRNAs and 15 rRNAs, while the genome of BH16-24 had 2039 genes 68 tRNAs and 18 rRNAs. Figure 3 shows the assembling results of BH15-2 and BH16-24 strains. The circular genomes of the two strains exhibited the coding sequence (CDS), repetitive sequences, GC content, number of RNA and GC skew (Fig. 3), where the outer 2 and 3 circles represented the CDS on the positive strand and negative stands. The genome sequences of BH15-2 and BH16-24 have been summited to the National Center for Biotechnology Information database with the accession number CP132229 and CP132230, respectively. Bioinformatics analysis indicated that BH15-2 and BH16-24 contains 71 and 21 unique genes, respectively, with a total of 2018 common genes.

**Table 3:** General features of BH15-2 and BH16-24.

Characteristic	BH15-2	BH16-24	Characteristic	BH15-2	BH16-24
Size (bp)	2,175,659	2,153,918	Gene cluster	3	3
GC%	36.80%	36.83%	Paralogy gene	4	4
Circular	circular	circular	tRNA	60	68
Gene number	2090	2039	rRNA	15	18
Gene total length (bp)	1,929,711	1,902,801	sRNA	8	8
Gene length/Genome (%)	88.70%	88.34%	Signal peptide	134	133
repetitive sequences	36,160 bp	36,204 bp	Transmembrane protein	523	520
CRISPR Number	9	8	Secreted protein	134	133

#### GIs analysis

**Fig. 4:**
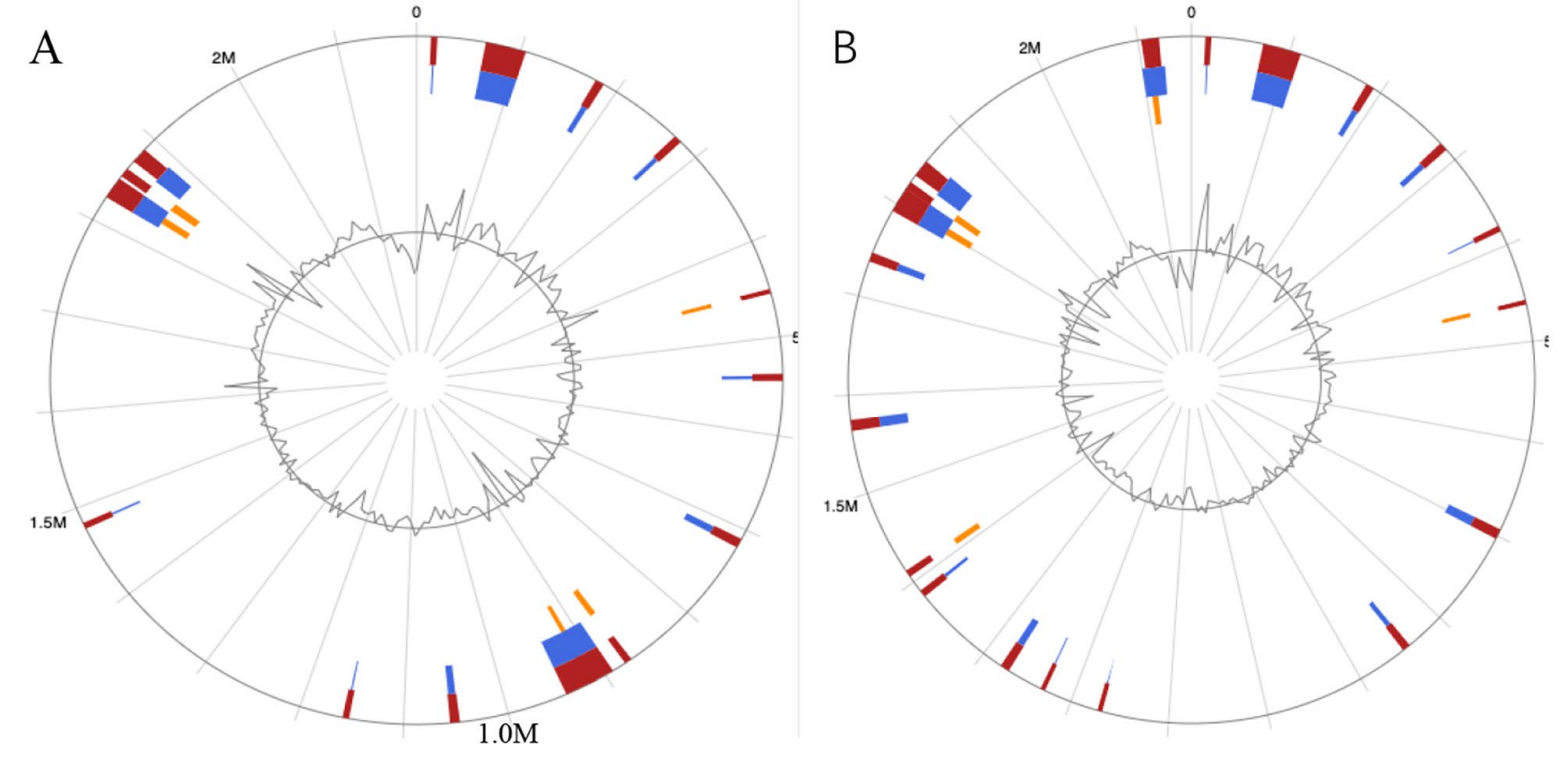
Circular visualization of the predicted Genomic Islands (GIs) on BH15-2 (**A**) and BH16-24 (**B**) strains. The analysis was conducted in IslandViewer 4. The interactive visualization of the distinct islands across the genomes is shown with blocks colored according to the predictor tool as described: red represents the predicted by at least one method, blue represents that the results of IslandPath-DIMOB predicted, yellow represents the predicted results of SIGI-HMM

The genomes of BH15-2 and BH16-24 were screened for horizontally acquired DNA using IslandPath-DIMOB, SIGI-HMM, IslandPick, and Islander methods integrated with the IslandViewer server (Fig. 4). In the genome of BH15-2, 17 presumed genome islands (GIs) ranging from 4912 to 50,177 bp were detected. The largest GI consisted of 50,177 bp with 79 predicted gene coding regions, of which 71 genes were unique in BH15-2 genome. And the shortest GI consisted of only 5 predicted gene. A total of 275 genes were predicted into GIs. For strain BH16-24, 21 presumed GIs ranging from 4716 to 38,833 bp were detected. The largest GI consisted of 38,833 bp and predicted to encode 45 genes. A total of 263 genes were predicted into GIs. It was found that most of the GIs of the two strains are the same by comparing the GIs of BH15-2 and BH16-24. However, based on the large inversion fragment between BH15-2 and BH16-24, the positions of several GIs are very different. The two strains each have a unique gene island, composed of unique genes (circled in red in Fig. 4). Both strains have no unique genes encoding virulence or drug resistance related genes.

#### Prophage analysis

**Fig. 5:**
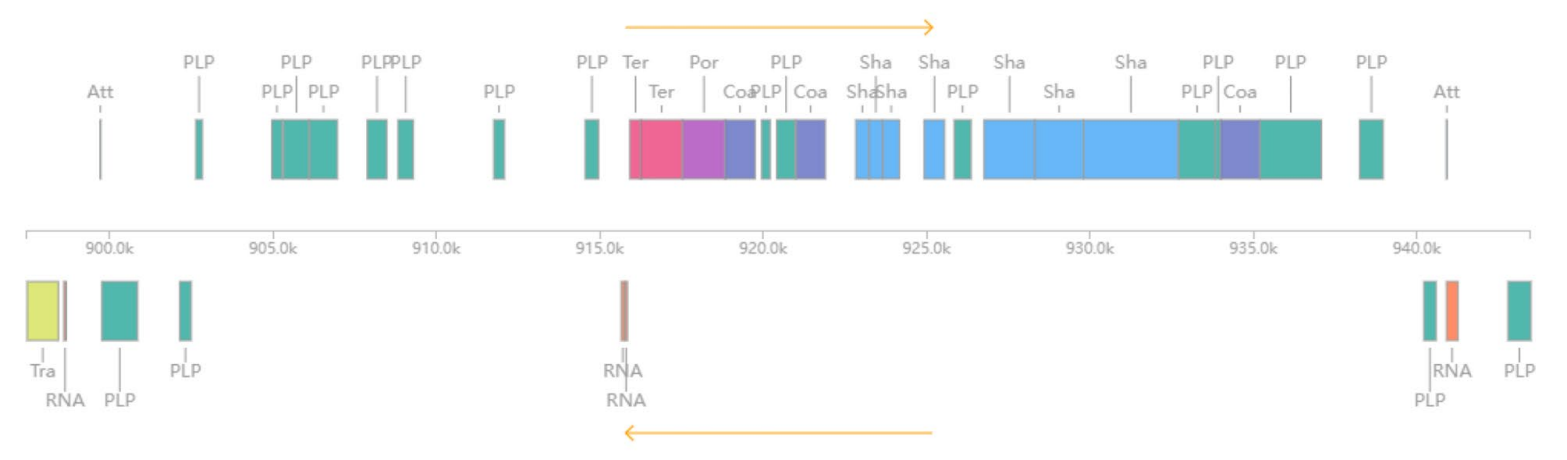
Genomic organization of coding sequences (CDS) of prophage in BH15-2 (show annotated only) Att: Attachment site; PLP: Phage-like protein; Ter: Terminase; Por: Portal protein; Sha: Tail shaft; Coa: Coat protein; Tra: Transposase

Prophage analysis of BH15-2 and BH16-24 showed that the two strains harbored 11 identical incomplete and questionable prophages (Supplementary Figure S1). But due to the inversion fragment, several of them are in different positions. Further, BH15-2 harbored an intact prophage and is also an active prophage predicted by Prophage Hunger (Fig. 5), which was absent in BH16-24. The intact prophage is 46 kb size located at 897,486 bp – 943,491 bp, contained a putative *attL* site and an *attR* site. And it encoded 60 prophage-related proteins and 17 hypothetical proteins, including phage tail protein, phage protein, siphovirus Gp157 family protein, ImmA/IrrE family metallo-endopeptidase, XRE family transcriptional regulator and endolysin.

#### Large-scale genomic inversion fragment

**Fig. 6:**
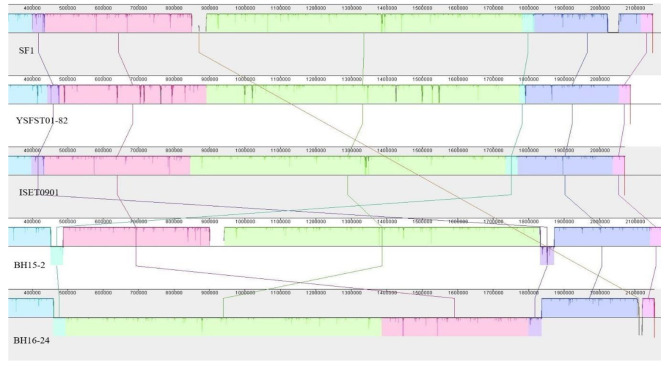
Mauve comparison diagrams of the BH15-2, BH16-24, SF1, YSFST01-82 and ISET0901. The scale represents the coordinates of each genome. Different color blocks represent local collinear blocks (LCBs), which are conserved segments in five genomes. Within LCBs, the white area represents low similarity regions or regions unique to one genome but absent in another. LCBs above the black horizontal central line are in forwarding orientation and below this are in reverse orientation. Colored lines show the rearrangement of LCBs among the genomes

The genomic synteny analysis of the whole *S.iniae* genome of BH15-2, BH16-24, and other three strains SF1, YSFST01-82 and ISET0901 which separately isolated from *Oreochromis niloticus*, and *Paralichthys olivaceus* was performed using Mauve software (The strain information is listed in Table 4). The result showed that all strains except BH16-24, were quite similar with respect to genome structure, with the exception of some small inversion. Thus, all strains except BH16-24 shared a similar synteny with each other. The BH16-24 genome displayed a large-scale inversion including 1.28 Mbp occurred across the origins/terminus axis (ori/ter axis), which located in the region from 502,513 bp-1,788,813 bp (Fig. 6).

**Table 4:** *S.iniae* genome sequences included in genomic comparison

Strain	Host	Location	Year	Genome status	Size	Accession number	References
ISET0901	*Oreochromis niloticus*	USA	2007	Complete	2.07	CP007586	[17]
YSFST01-82	*Paralichthys olivaceus*	South Korea	─	Complete	2.09	CP010783	[18]
SF1	*Paralichthys olivaceus*	China	2006	Complete	2.15	CP005941	[1]
BH15-2	*T.ovatus*	China	2015	Complete	2.18	CP132229	This study
BH16-24	*T.ovatus*	China	2016	Complete	2.15	CP132230	This study

#### Antibiotic resistance genes in the inversion fragment

The large inversion fragment encodes 1242 genes. Among them, many antibiotic resistance genes including efflux system genes, ABC transporter and β-lactamase-encoding were screened out, such as penicillin-binding protein 2B (*pbp2B*), penicillin-binding protein 1 A (*pbp1A*), penicillin-binding protein 2X (*pbp2X*), aminoacyltransferase (*femA*), DNA gyrase subunit A (*gyrA*), oligopeptide-binding protein (*oppA*), aminoglycoside phosphotransferase (*aph*), dihydrofolate reductase (*dfrA*), macrolide export ATP-binding/permease protein (*macB*) (Table 5).

**Table 5:** The main antibiotic resistance genes encoded by the large inversion fragment

Gene ID of BH15-2	Locus	Gene ID of BH16-24	Locus	Gene name	Annotation
GE000496	524382:525752:	GE001692	1758762:1760132:+	─	MATE family efflux transporter
GE000551	577162:578196:+	GE001637	1705598:1707352:	*exp8*	putative ABC transporter ATP-binding protein exp8
GE000596	619779:621863:+	GE001592	1662650:1664734:	*pbp2B*	penicillin-binding protein 2B
GE000620	647867:649018:+	GE001568	1635495:1636646:	─	MFS transporter
GE000700	737844:739064:+	GE001488	1545449:1546669:	─	MFS transporter Putative metabolite transport protein HI_1104
GE001036	1056918:1058150:+	GE001224	1267295:1268527:	*femA*	aminoacyltransferase
GE001062	1080994:1083471:	GE001198	1241974:1244451:+	*gyrA*	DNA gyrase subunit A
GE001142	1160475:1160972:	GE001118	1164473:1164970:+	*dfrA*	dihydrofolate reductase
GE001150	1168472:1170214:	GE001110	1155231:1156973:+	*yheH*	probable multidrug resistance ABC transporter ATP-binding/permease protein YheH
GE001151	1170216:1171937:	GE001109	1153508:1155229:+	*yheI*	probable multidrug resistance ABC transporter ATP-binding/permease protein YheI
GE001155	1176552:1178318:	GE001105	1147127:1148893:+	─	uncharacterized ABC transporter ATP-binding protein TM_0288
GE001156	1178318:1180060:	GE001104	1145385:1147127:+	─	uncharacterized ABC transporter ATP-binding protein MT1311
GE001179	1200176:1201420	GE001081	1124025:1125269:+	*macB*	macrolide export ATP-binding/permease protein
GE001181	1202164:1203408:	GE001079	1122037:1123281:+	─	HlyD family efflux transporter periplasmic adaptor subunit
GE001242	1265692:1265880	GE001018	1059565:1059753:+	*rpoB*	DNA-directed RNA polymerase subunit beta
GE001265	1293282:1294205:	GE000995	1031240:1032163:+	*lnrL*	linearmycin resistance ATP-binding protein LnrL
GE001278	1305545:1307497:	GE000982	1017948:1019900:+	*gyrB*	DNA gyrase subunit B
GE001318	1347610:1348833:+	GE000942	976612:977835:	*femA*	aminoacyltransferase
GE001353	1388065:1389714:	GE000907	935731:937380:+	*oppA*	oligopeptide-binding protein
GE001395	1423485:1424108:	GE000865	901337:901960:+	*macB*	macrolide export ATP-binding/permease protein
GE001541	1566232:1567062:	GE000719	758383:759213:+	*Rv1218c*	multidrug efflux system ATP-binding protein Rv1218c
GE001634	1654361:1656568:+	GE000626	668874:671081:	*pbp 1 A*	penicillin-binding protein 1 A
GE001646	1668281:1670533:	GE000614	654909:657161:+	*pbp 2X*	penicillin-binding protein 2X
GE001685	1714423:1715214:	GE000575	610228:611019:+	*aph*	aminoglycoside phosphotransferase
GE001686	1715275:1716309:	GE000574	609133:610167:+	*ecsB*	multidrug ABC transporter permease

#### Virulence related genes in the inversion fragment

According to the annotation results of the VFDB (Virulence Factors of Pathogenic Bacteria) database, the two strains contain 336 putative virulence related genes, of which 221 virulence genes are in the inversion fragment. Among them, several important virulence factor such as capsule (*cpsA*, *cpsB*, *cpsC*, *cpsD*), CAMP factor (*cfa/cfb*), C5a peptidase (*scpA*, *scpB*, *scpI*), phosphoglucomutase (*pgmA*), beta-hemolysin/cytolysin (*cylG*, *cylA*, *cylA*, *cyll*), hemolysin A/B (*hlyA*, *hlyB*), laminin-binding protein (*lmb*), arein the inversion fragment (Table 6).

**Table 6:** The main virulence genes encoded by the large inversion fragment

Gene ID of BH15-2	Locus	Gene ID of BH16-24	Locus	Gene name	Virulence_factor_name
GE000743	786971:787735:+	GE001445	1496778:1497542:	*cylG*	Beta-hemolysin/cytolysin
GE000813	862854:864317:+	GE001375	1419932:1424935:	*cpsA*	Capsule
GE000814	864314:865045:+	GE001374	1419204:1419935:	*cpsB*	Capsule
GE000815	865054:865743:+	GE001373	1418506:1419195:	*cpsC*	Capsule
GE000816	865754:866473:+	GE001372	1417776:1418495:	*cpsD*	Capsule
GE000817	866518:868287:+	GE001371	1415962:1417731:	*cpsE*	Capsule
GE000818	868290:868892:+	GE001370	1415357:1415959:	*cps4E*	Capsule
GE000928	949943:950500:+	GE001332	1374945:1375502	*sipA*	PI-2
GE000938	961136:961906:+	GE001322	1363539:1364309:	*cfa/cfb*	CAMP factor
GE000985	1004254:1007622:+	GE001275	1317823:1321191:	*scpI*	C5a peptidase
GE000986	1008037:1009752:	GE001274	1315693:1317408:	*pgmA*	Phosphoglucomutase
GE001008	1031614:1032162:	GE001252	1198492:1200039:	*cap8J*	capsular
GE001087	1105346:1106104:	GE001173	1219341:1220099:+	*cylG*	Beta-hemolysin/cytolysin
GE001109	1127171:1131715:	GE001151	1193730:1198274:+	*scpB*	C5a peptidase
GE001118	1140851:1141744:	GE001142	1183701:1184594:+	*cpsG*	Capsule
GE001158	1180643:1182529:	GE001102	1142916:1144802:+	*hlyB*	Hemolysin B
GE001193	1212225:1213421:	GE001067	1112024:113220:+	*capA*	Capsule
GE001226	1244626:1245480:	GE001034	1079965:1080819:+	*cap8N*	Capsule
GE001245	1267962:1269368:	GE001015	1053562:1055567:+	*yscN*	T3SS
GE001265	1293282:1294205:	GE000995	1031240:1032163:+	*cylA*	Beta-hemolysin/cytolysin
GE001296	1321060:1322616:	GE000964	1002829:1004385:+	*lmb*	Laminin-binding protein
GE001473	1497597:1500002:	GE000787	825443:827848:+	*essC*	T7SS
GE001493	1518678:1523573:	GE000767	801872:806767:+	*scpA*	C5a peptidase
GE001536	1561529:1562863:	GE000724	762582:763916:+	*hlyA*	Hemolysin A
GE001652	1675665:1676399:	GE000608	649043:649777:+	*cylA*	Beta-hemolysin/cytolysin
GE001690	1718090:1719385:	GE000570	606057:607352:+	*cps4A*	Capsule
GE001707	1733970:1735202:	GE000553	590240:591472:+	*cyll*	Beta-hemolysin/cytolysin
GE001708	1735217:1735951:	GE000552	589491:590225:+	*cylG*	Beta-hemolysin/cytolysin

## Discussion

There have been many studies to distinguish the genotypes of *S.iniae* using molecular methods. However, the multidrug resistance and virulence differentiation characters and the underlying mechanisms with different genotypes have been poorly explored to date. The investigation on the molecular mechanisms of drug resistance and virulence variation is essential to the prevention of further spreading of these multidrug resistance strains or the occurrence of new resistant strains. The results showed great significance to explain the multidrug resistant and virulence differentiation of *S.iniae* strains. As reflected by our results, BH16-24 is a native multidrug resistant and low virulence strain. Further genomic comparisons between BH15-2 and BH16-24 revealed valuable information on the possible multidrug resistance and virulence differentiation among *S.iniae* strains. To our knowledge, this is the first report on the differences in biological characteristics and genomes among different genotypes of *S.iniae*.

Fish infected of *S.iniae* often show a variety of clinical signs, such as anorexia, lethargy, erratic swimming, and visceral hemorrhage [19]. Similarly in the present study, clinical signs including meningoencephalitis, enteritis, hemorrhage of pterygiophore, operculum, liver hemorrhage were also observed. In our previous study, the two genotypes strains of *S.iniae* showed identical phenotypic features [16]. However, the growth ability of the two strains was different, where the growth rate of BH16-24 was significantly faster than that of BH15-2 at the exponential stage according to the growth curve.

*S.iniae* recovered from farmed fish were genetically distinct from wild reef fish and exhibited a trend toward higher minimal inhibitory concentrations against several antibiotics [20]. Consistently, our results suggest that BH15-2 was susceptible to most of the tested antibiotics except neomycin and gentamycin. In contrast, BH16-24 exhibited multidrug resistance, including high resistance against penicillin, ceftazidime, sulfasomizole, compound sulfamethoxazole, spectinomycin, polymyxin B, rifampin and intermediate against ceftriaxone sodium, ampicillin, cefazolin, neomycin, and gentamycin. In the previous study, *S.iniae* obtained from fish were susceptible to most of the antibiotics [4, 21]. However, according to recent reports, more and more resistant strains of *S.iniae* was found to be resistant to many important antibiotics including amoxicillin, penicillin, ampicillin, gentamycin,spectinomycin, amikacin, neomycin, enrofloxacin, lincomycin, and sulfamethoxazole [17, 22, 23]. The emergence of multidrug resistance strain is worrisome. In addition, both BH15-2 and BH16-24 remained highly susceptible to tetracycline, erythromycin, norfloxacin, azithromycin, ciprofloxacin, chloramphenicol and deoxytetracycline, which is indicative that these antibiotics might be useful in controlling the disease in the future.

The relationship between genotype and virulence of *S.iniae* isolates was investigated. Fuller et al. reported that *S.iniae* virulence is associated with distinct genetic profile and demonstrated differences between pathogenic and nonpathogenic isolates [10]. The isolates belong to genotype 1 in rep-PCR analysis showed a high virulence in the flounder, while the isolated belonging to genotype 2 were relatively low in virulence [9]. Similarly, among 14 isolates of *S.iniae* with six clonal patterns, two clones have one fold lower in pathogenicity challenge than others [11]. 11 *S.iniae* isolates from diseased wild and farmed fish showed significant differences in virulence and persistence, with a certain correlation to genogroup [24]. In this study, the pathogenicity of the two strains were compared by intraperitoneal injection in *T.ovatus*. Similar results were obtained in our analyses, where the virulence of genotype 1 strain BH15-2 showed much greater virulence than that of genotype 2 strain BH16-24.

Genetic variability not only depends on point mutations but also largely on horizontal genes transfer and intra-genomic rearrangements, which may disrupt chromosome organization [25]. During cell division, the symmetry of the origin and terminus loci play a role in the precise choreography of replicated chromosome separation [26]. Genome arrangement may affect gene expression and is thought to be related to the diversity of phenotypes seen in organisms [27]. Studies have shown that after the common ancestor branch of *Streptococcus*, chromosome reversal across the replication axis often occurs in a single streptococcal species [28]. A previously study demonstrated that unbalanced genome is prone to generate DNA rearrangements in the M3 strain of *S.pyogenes*, which is caused by the loss or acquisition of phages [29]. Moreover, results from a previous study suggest that a large-scale genomic rearrangement may resulted in biological discrepancies between a native avirulent and highly virulent *S.suis* strains [30]. Compared with its parental strain GX005, YM011 had a 0.4 M large inversion fragment which may result in abnormal expression of some genes including drug resistance genes and virulence factors, eventually leads to virulence attenuation [31]. By comparing the whole genomes of the two strains with other *S.iniae* which were also isolated from diseases fish, a large-scale inverted fragment about 1.28 Mbp was found as the major difference in BH16-24. The genomic recombination in BH16-24 leads to abnormal expression of some resistance and virulence genes in the fragment, eventually leads to multidrug resistance and virulence attenuation. Thus, our findings are consistent with the notion that inversion events of intergenic regions correlate to phenotypic variation [32].

β-lactam antibiotics are commonly being used in aquaculture in some parts of the world to treat bacterial infections [33]. Penicillin-binding proteins (PBPs) are membrane proteins involved in the biosynthesis of peptidoglycans in bacterial cell walls. The β-lactam antibiotics participate in the synthesis of peptidoglycans by binding to the active site of PBPs, thereby disrupting the formation of normal cell walls and inducing cell death through bacteriolysis [34]. The major PBPs causing for the β-lactam antibiotics resistance of *S. pneumoniae* are PBP2x, PBP2b and PBP1a [35]. In our study, BH16-24 was resistant to the two β-lactam antibiotics i.e., penicillin, ceftazidime, and intermediate resistant toward the other three β-lactam antibiotics used in this study, i.e., ampicillin, cefazolin, ceftriaxone sodium, whereas BH15-2 was susceptibility to these antibiotics. Pbp1a, pbp2x, and pbp2b gene of BH16-24 is located on an inverted fragment, and its abnormal expression changes the amount of PBP protein, which may lead to BH16-24 resistance to β-lactam antibiotics. In addition, the present of the RNA polymerase beta subunit (*rpoB*) with mutations have been reported in rifamycin resistance in *Brucella melitensis*, *Mycobacterium tuberculosis*, and other microorganisms [36, 37]. The DNA gyrase (*gyrA*) have been suggested to be involved in fluoroquinolone resistant isolates of *S.agalatiae* [38]. The dihydrofolate reductase enzyme encoded by the *dfr* gene promotes bacterial resistance to trimethoprim [39, 40]. Aminoglycoside antibiotics are transported by the oligopeptide transport system, thus when the gene for *oppA* protein was deleted, sensitivity to aminoglycoside antibiotics was greatly decreased [41]. Aminoglycoside phosphotransferases which encoded by *aph* gene are bacterial enzymes responsible for the inactivation of aminoglycoside antibiotics by O-phosphorylation [42]. In this study, genes such as *gryA*, *gryB*, *rpoB*, *dfrA*, *oppA*, *aph* were in the inverted fragment, which may be related to the resistance of BH16-24 to sulfonamide antibiotics, aminoglycoside antibiotic, rifampin. Moreover, the efflux pumps play an important role in conferring resistance by actively excreting the harmful antibiotic drugs from the bacteria [43]. Efflux pumps exists in almost all bacterial species. It can not only excrete a wide range of antibiotics, but also reduce intracellular antibiotic concentration and promote mutation accumulation [43, 44]. Here, we identified several efflux-pump related genes from different efflux-pump families among the genomes. The abnormal expression of these resistance genes on the inverted fragment may lead to multiple drug resistance of BH16-24.

To date, several virulence-associated factors (VAFs) have been characterized which are closely related to the pathogenesis of *S.iniae* infection. The most critical VAF validated in *S.iniae* including polysaccharide capsular (*cps*), phosphoglucomutase (*pgmA*), M-like protein (*simA*), beta-hemolysin/cytolysin (*cyl*), C5a peptidase (*scp*). CPS of *S.iniae* is a major virulence factor that provides resistance to the bactericidal activity of phagocytes and stimulates prolonged inflammatory responses, which including *cpsA*, *cpsB*, *cpsC*, *cpsD*, *cpsE*, *cpsG*, *cpsJ* [45, 46]. The streptococcal CpsA protein was reported to associated with important virulence determinants, including cell wall processing [47], polysaccharide synthesis [48], and reaction to antimicrobial stress [49]. The absence of *cpsD* could reduce the ability of *S.iniae* to survive phagocytosis and escape the immune system [50]. CpsJ influences the synthesis of CPS and loss of this protein showed lower virulence in a channel catfish infection model [51]. The *pgm* gene play an pivotal role in normal cell wall morphology, surface capsule expression, resistance to innate immune clearance mechanisms, therefore it is necessary for the virulence in *S.iniae* [52]. M-like protein contributes to cellular adherence and invasion and provides resistance to phagocytic killing based in vitro cell analysis [53]. The virulence factor Beta-hemolysin/cytolysin is encoded by *cyl* gene, capable of exerting cytolytic, proapoptotic, proinvasive, proinflammatory, or antiphagocytic effects on a variety of target cells [54]. In our study, both genomes harbor 336 putative genes involved in virulence, among which 221 virulence genes are located on the inversion fragment. Among the 221 virulence genes, we observed genes encoding CPS, phosphoglucomutase, M-like protein, beta-hemolysin/cytolysin, C5a peptidase. The abnormal expression of these virulence-related genes on the inverted fragments contributed to the reduced virulence of BH16-24.

Prophage can enhance bacterial adherence to animal cells, encode a series of bacterial toxins, and affect bacterial biofilm formation, which is closely related to bacterial virulence [55–57]. As reported by Wang et al., the phage was the major reason of causing different levels of virulence between *S.agalactiae* strains [58]. Prophage analysis of BH15-2 and BH16-24 showed that both strains harbored 11 identical incomplete and questionable prophages, although several of them are in different positions due to the inversion fragment. Moreover, there is a 46 kb length, intact phage sequence which only existed in the BH15-2 genome. The GC contents of the prophage fragment (35.46%) deviate from the host genomes (36.80%). The prophage encoded 77 proteins, and 17 genes encoded hypothetical proteins, the other 60 genes encoded phage hit proteins such as phage lysin, phage tail protein, and phage integrase. However, the function of the prophage in BH15-2 is still unknown, and whether it is related to its virulence needs further experiments to verify.

## Conclusions

In summary, we compared the biological characteristics such as growth, virulence, drug resistance, and whole genome sequence of two different genotypes of *S.iniae.* Comparative genomic studies of BH15-2 and BH16-24 showed that the main difference is a 1.28 Mbp inversion fragment. The inversion fragment may lead to abnormal expression of drug resistant and virulence genes, which is believed to be the main reason for the multiple resistance and weakened virulence of BH16-24. Aside from the differences in genomic rearrangement, BH15-2 harbored a novel intact prophage which is absented in BH16-24. There was finished concordance between genotypic evidence and biological characteristics. Further research is needed on how the genomic rearrangements affect the gene expression, drug resistance, and pathogenicity of *S.iniae*.

## Materials and methods

### Bacterial strains

The *S.iniae* strain BH15-2 and BH16-24 were originally isolated from the livers of moribund cultured golden pompano on two separate farms of China in 2015 ang 2016, respectively [16]. The fish from the two outbreaks displayed similar clinical signals, and the cumulative mortality rate was approximately 20–30%. Briefly, the stored strains were removed from − 80 ℃ refrigerator and streaked onto the BHI plate, cultured at 30 ℃ for 24–48 h. Then picked up a single colony and inoculated into 10 ml of BHI medium, cultivated at 30 ℃ by shaking.

### Growth analysis

To measure the growth level of bacteria in BHI broth, overnight cultures of BH15-2 and BH16-24 were inoculated into BHI with an initial OD_600_ of 0.01 in a ratio of 1:50, respectively. The cultures were collected every 2 h of intervals and the optical density was measured at 600 nm from 0 to 18 h of growth at 30 ℃ with shaking in 180 r/min. Data were expressed as mean ± standard deviation (SD). Statistical analyses were performed using Student’s t-test using SPSS 21.0 and *p* < 0.05 was considered significant.

### Comparison of the virulence between BH15-2 and BH16-24

Adult golden pompano with a mean weight of 50 g, were purchased from a local fish farm and maintained in a 16 m^3^ tank with aeration and sand-filtered seawater supply. The fish (n = 150) were acclimated for 2 weeks at 28–30 ℃ and checked randomly to confirm that no bacterial infected. Fish were fed twice daily with commercial fish expanded pellets (Guangdong Yuehai Feed Group), and waste was removed daily. The bacterial concentration determined by plating 10-fold serial dilutions onto BHI agar plates. Suspensions from 1.5 × 10^8^ to 1.5 × 10^5^ CFU/ml were prepared by serial 10-fold dilution. Fish were divided into nine groups with 16 fish per group. Before experimental treatment or organ extraction, fish were euthanized in 100 mg/L MS-222 (Sigma, USA). Eight groups were injected intraperitoneally (i. p.) with 0.1 ml of diluted bacterial cell suspension of the strain BH15-2 and BH16-24 at the final concentration of 1.5 × 10^8^, 1.5 × 10^7^, 1.5 × 10^6^ and 1.5 × 10^5^ CFU/ml, respectively. The control group were i. p. with the same amount of sterilized saline. The mortalities were recorded every 24 h interval for 28 days post-infection. The bacteria were reisolated from the brain, kidney and spleen tissues of all dead fishes at the end of the experiment and identified.

### Comparison of the antibiogram between BH15-2 and BH16-24

The antibiogram study of the bacterium was determined on BHI plates according to the disc diffusion method, and the diameters of the inhibition zones were measured using Vernier calipers. The tested antibiotic impregnated discs were summarized in Table 2. Resistant, intermediate, and susceptible phenotype determinations were based on manufacturer guidelines (Hangzhou Binhe Microorganism Reagent Co., Ltd., China). Multiple antibiotic resistance (MAR) index of the two strains against the tested antibiotics was calculated by following the procedure described by Krumperman [59].

### Genome sequencing and annotation

The genomes of BH15-2 and BH16-24 were sequenced by PacBio sequencing at the Beijing Biomarker Bioinformatics Technology Co., Ltd. For genome assembly, the filtered subreads were assembled by Canu v1.5 software [60], and then circlator v1.5.5 was taken to cyclizing assembly genome [61].

### Genome component prediction

For genome component prediction, coding genes prediction was performed by Prodigal v2.6.3 [62]. The GenBlastA v1.0.4 program was used to scan the whole genomes after masking predicted functional genes [63]. Putative candidates were then analyzed by searching for non-mature mutations and frame-shift mutations using GeneWise v2.2.0 [64]. Transfer RNA (tRNA) genes were predicted with tRNAscan-SE v2.0 [65], Ribosome RNA (rRNA) genes were predicted with Infernal v1.1.3 [66]. Repetitive sequences were predicted using RepeatMasker v4.0.5 [67]. CRT v1.2 was used for CRISPR identification [68]. Circos v0.66 was used to draw genomic circles [69].

### Gene functions

For functional annotation, the predicted proteins were blast (e-value: 1e^− 5^) against Nr (Non-Redundant Protein Database databases), Swiss-Prot, TrEMBL, KEGG (Kyoto Encyclopedia of Genes and Genomes), eggNOG, GO (Gene ontology). The pathogenicity and drug resistance of pathogenic bacteria were analyzed using VFDB and ARDB (Antibiotic Resistance Genes Database).

### Comparative genomics analysis

Genomic synteny was analyzed using Mauve v2.3.1 [70]. GIs of BH15-2 and BH16-24 were determined with IslandViewer 4 [71]. PHASTER was used to identify prophage sequences [72]. Prophage Hunger was used to predict the intact prophage [73].

### Electronic supplementary material

Below is the link to the electronic supplementary material.

Supplementary Figure 1: Circular visualization of the predicted prophage on BH15-2 (**A**) and BH16-24 (**B**) strains. The analysis was conducted in PHASTER. The interactive visualization of the distinct prophage across the genomes is shown with blocks colored according to the predictor tool as described: red represents incomplete prophages, blue represents questionable prophages, green represents intact prophages

## Data Availability

The whole-genome sequence data of BH15-2 and BH16-24 have been deposited at the National Center for Biotechnology Information database with the accession number CP132229 and CP132230.

## Abbreviations

RAPD: Random amplified polymorphic DNA
rep-PCR: Repetitive primer polymerase chain reaction
BHI: Brain heart infusion
i. p.: Injected intraperitoneally
Dpi: Days post-infection
MDR: Multidrug-resistant
CDS: Coding sequence
LCBs: Local collinear blocks
VFDB: Virulence Factors of Pathogenic Bacteria
ARDB: Antibiotic Resistance Genes Database
Nr: Non-Redundant Protein Database databases
KEGG: Kyoto Encyclopedia of Genes and Genome
GO: Gene ontology
ori/ter axis: Origins/terminus axis
GIs: Gene Islands
PBPs: Penicillin-binding proteins
SD: Standard deviation
