# Supplementary material for: Comparative genomics analysis of Streptococcus iniae isolated from Trachinotus ovatus: novel insight into antimicrobial resistance and virulence differentiation
Source: BMC Genomics. 2023 Dec 14;24:775. doi: 10.1186/s12864-023-09882-5 (PMC10720119; doi:10.1186/s12864-023-09882-5)
Supplement: Supplementary file 1 — Supplementary Figure 1: Circular visualization of the predicted prophage on BH15-2 (A) and BH16-24 (B) strains. The analysis was conducted in PHASTER. The interactive visualization of the distinct prophage across the genomes is shown with blocks colored according to the predictor tool as described: red represents incomplete prophages, blue represents questionable prophages, green represents intact prophages [file 12864_2023_9882_MOESM1_ESM.docx]

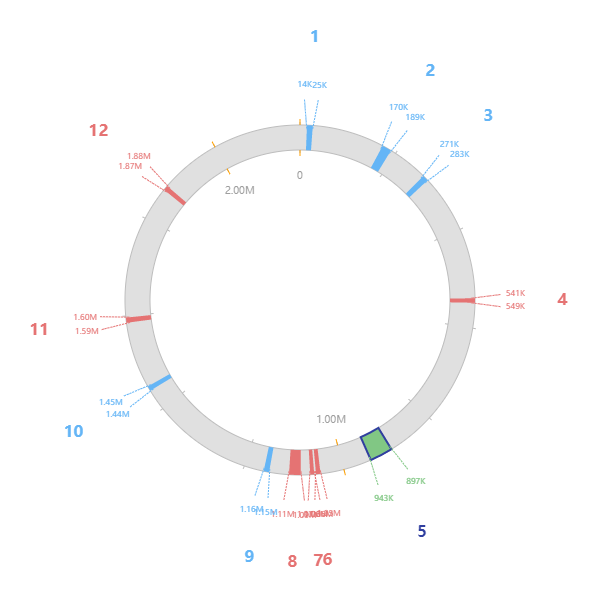

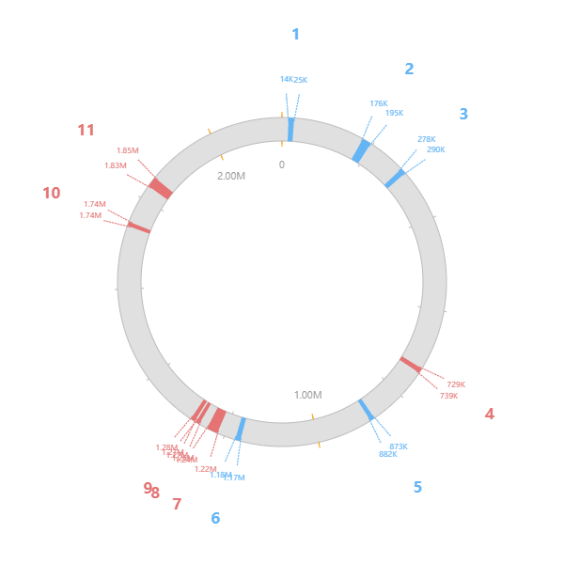


B

A

Supplementary Figure 1 Circular visualization of the predicted prophage on BH15-2 (A) and BH16-24 (B) strains. The analysis was conducted in PHASTER. The interactive visualization of the distinct prophage across the genomes is shown with blocks colored according to the predictor tool as described: red represents incomplete prophages, blue represents questionable prophages, green represents intact prophages.
